# Cross-cultural adaptation, reliability, and validity of the Turkish version of moral courage scale for physicians

**DOI:** 10.1371/journal.pone.0333598

**Published:** 2025-10-30

**Authors:** Şerife Yılmaz, Gamze Özbek Güven, Feyza İnceoğlu

**Affiliations:** 1 Harran University Faculty of Medicine History of Medicine and Ethics Department, Şanlıurfa, Türkiye; 2 Yüksek Ihtisas University Faculty of Medicine History of Medicine and Ethics Department, Ankara, Türkiye; 3 Departmant of Bioistatistic, Turgut Özal University Faculty of Medicine, Malatya, Türkiye; Ajman University, UNITED ARAB EMIRATES

## Abstract

**Aim:**

Physicians, like other healthcare professionals, frequently encounter situations requiring moral courage in their professional lives. However, studies on the moral courage of physicians are limited in the literature. This study aims to evaluate the Turkish adaptation, validity, and reliability of the Moral Courage Scale for Physicians (MCSP) and to contribute to national and international studies on moral courage among physicians.

**Materials and methods:**

This methodological study involved the adaptation, validation, and reliability assessment of the MCSP in Turkish. The scale’s internal consistency was evaluated using Cronbach’s α coefficient, and the total correlation coefficients for the items were calculated. Test-retest reliability was assessed using a two-group design.

**Results:**

The Turkish version of the MCSP demonstrated high internal consistency, with a Cronbach’s α coefficient of 0.91. The total correlation coefficients for the items ranged from 0.387 to 0.797. A significant positive correlation was found between the test and retest scores of the MCSP (p < 0.05).

**Conclusion:**

The Turkish version of the MCSP is a valid and reliable tool for assessing moral courage among physicians.

## Introduction

Moral courage is a concept that represents an individual’s strength to act in accordance with moral and ethical values [1]. It involves the ability to defend what is right despite potential negative consequences [2] and reflects the inner strength to uphold one’s moral and ethical values in the face of threats [3].

From a healthcare and medical perspective, moral courage includes the ability to advocate for the needs of patients, protect their treatment rights, and intervene in patient care. This is critically important in terms of being committed to acting in the best interests of patients. Moral courage requires helping patients confront their vulnerabilities and pains, witnessing their vulnerabilities and pains, and acting with determination and dedication to provide professional care [2,4–7]. In this context, the demonstration of moral courage by healthcare professionals can contribute to gaining patients’ trust, acting in accordance with ethical standards, and protecting individuals’ rights in the process of receiving healthcare.

Moral courage holds great value for healthcare personnel. However, the demonstration of moral courage is influenced not only by an individual’s internal values but also by environmental factors. Factors such as working conditions, role conflicts, power dynamics, and organizational culture in the healthcare sector are significant barriers that individuals face when demonstrating moral courage. Additionally, demonstrating moral courage can sometimes expose individuals to negative reactions, intimidation, and even the risk of psychological or physical violence from colleagues or superiors. These situations imply the risk of individuals losing their jobs or status. Personal or organizational barriers can lead healthcare personnel to struggle to act in accordance with ethical values and experience traumatic experiences despite knowing what is right [8].

The lack of moral courage can have potentially significant consequences for both healthcare personnel and patients [9]. For healthcare personnel, a lack of moral courage can lead to difficulties in making ethical decisions or behaving in accordance with moral values. This can lead to healthcare personnel having difficulty coping with moral stress, which can affect their professional satisfaction and performance. For patients, a lack of moral courage can pave the way for unethical behaviors or negative events. When healthcare personnel struggle to uphold ethical principles, patients’ rights to respect and receive treatment in accordance with ethical standards may be at risk. This can lead to patients losing their trust and negatively impacting their treatment processes This situation may, at times, give rise to manifestations of violence in healthcare. Violence against physicians and other healthcare professionals is not only a phenomenon that threatens the safety and well-being of individuals, but also constitutes a serious violation of the fundamental ethical principles underlying medical practice, such as nonmaleficence, justice, and respect for persons. In such circumstances, preserving professional integrity, continuing to prioritize patient welfare, and collectively standing against violence represent one of the most concrete expressions of moral courage for healthcare professionals. Refusing to remain silent in the face of violence, supporting colleagues, and striving to raise public awareness are ethical responsibilities and acts of moral courage required at both the individual and institutional levels [10]. Medical educators should continue to demonstrate and emphasize examples of moral courage in medical practice and encourage trainees to do the right thing despite potential negative outcomes [11].

Physicians often encounter ethical challenges in their professional practice. One of the important qualities that physicians need to overcome such challenges is courage [12]. Various strategies are implemented during medical education to develop physicians’ moral courage. These strategies include discussing ethical principles, case studies, and role-playing activities. However, there is limited evidence regarding the effectiveness of these strategies, partly due to the difficulty in effectively measuring physicians’ moral courage [13].

Recent extraordinary events, both national (such as devastating earthquakes affecting many provinces in Türkiye) and global (such as the Coronavirus pandemic), have strained the healthcare system and exposed physicians to ethical conflicts. Assessing the levels of physicians’ moral courage following such events is crucial for understanding the impact of these challenges.

Studies on moral courage in healthcare professionals generally focus on nurses, and research on the moral courage of physicians is limited. In the Turkish literature, the lack of a scale to evaluate the level of moral courage of physicians highlights this gap. In this context, a comprehensive study is aimed to be conducted by conducting a Turkish validity and reliability study of the MCSP developed by Martinez and colleagues (2016), in order to understand cultural differences in this field [13].

## Materials and methods

### Study design and sample

The Turkish adaptation of the Moral Courage Scale was conducted as a methodological study. The sample size to be used in the study was determined taking into account the number of items in the scale. It was planned to include at least 10 times the number of items in the scale (9) as sampling units in the study [14], and the study was completed with 106 participants. Participants were selected using non-probabilistic sampling methods, specifically volunteer sampling and snowball sampling. The study was conducted between April 25, 2022, and December 30, 2022.

Anderson and Gerbing (1984) stated that when there are three or more items per factor, a sample size of 100 is generally sufficient for convergence. Similarly, MacCallum et al. (1999) argued that power analysis is not typically recommended for factor analysis since it is considered an exploratory process, and that a minimum of 100 observations can be regarded as adequate for such analyses [15,16].

### Ethical considerations

The study was conducted in accordance with the Helsinki Declaration and was approved by the Duzce University Non-Interventional Ethics Committee (Ref: 2022/80). Participants were thoroughly informed about the purpose and procedures of the study. Ensuring the confidentiality and privacy of their personal information was prioritized, and all data were handled with strict confidentiality. Before starting the survey, respondents were informed about the aim of the study, anonymity, and measures of data protection and gave their consent to participate by clicking the respective field.

### Data analysis

Data analysis was conducted using SPSS 26.0 for descriptive factor analyses, descriptive statistics, and reliability assessments, while AMOS 24 was employed for confirmatory factor analysis. A reliability coefficient threshold of 0.95 and a significance level of 0.05 were used in the analyses. Descriptive statistics were presented as mean ± standard deviation for quantitative data and as frequencies and percentages for qualitative data.

### Language validity

Following permission to adapt the “Moral Courage Scale” by Martinez (2016) into Turkish, a blind translation was performed by two bilingual experts aware of the study’s purpose but not its content. The two translated versions were compared, and the items that best aligned with the original scale were selected. Subsequently, a back-translation was conducted by a native English speaker fluent in Turkish, and this version was compared with the original. A 9-item questionnaire pool was finalized [17].

### Content (expert) validity

The scale was reviewed by a panel of 12 experts with specialized knowledge in medical history, ethics, and nursing ethics. Of these, three experts were specialists in nursing ethics and nine in medical ethics. Experts were selected based on their academic background, professional experience, and recognized expertise in their respective fields. Each expert independently evaluated the clarity and necessity of each item using a 3-point Likert-type scale (1 = not necessary, 2 = useful but not sufficient, 3 = necessary and should be included), ensuring a systematic assessment of content validity. The content validity index was calculated based on their feedback. The Kendall’s W value was 0.029, which was not statistically significant (χ² = 2.737, p = 0.950). It was concluded that the 9 items were understandable and appropriate for the Turkish context, allowing the study to proceed to the pilot stage [18,19].

The Content Validity Index (CVI) was calculated based on expert opinions and is presented in Table 1.

**Table 1:** Content Validity Index (CVI) results.

Madde	I-CVI
Q1	0.92
Q2	0.92
Q3	0.92
Q4	0.92
Q5	1.00
Q6	0.92
Q7	0.83
Q8	0.92
Q9	0.83
S-CVI/Average	0.91

An I-CVI value above 0.78 and an S-CVI/Average value above 0.90 indicate that the item is considered appropriate by experts. Examination of the scale items revealed that the item-level I-CVI values were at an adequate level. The S-CVI/Average value was found to be 0.91 [20].

### Pilot study

A pilot application of the Moral Courage Scale, consisting of 9 items, was conducted with 20 individuals. A one-week period at the beginning of the study was allocated for the pilot implementation, which was conducted between April 25, 2022, and May 4, 2022. When the responses to the items were examined, it was observed that there were no difficulties in understanding, the responses were given easily, and the questions were suitable for the population. Therefore, data collection for the scale was initiated.

### Psychometric testing of the moral courage scale

During the data collection process, 106 participants were reached, and the collected data were prepared for analysis. Variance homogeneity, normal distribution, independence, randomness, and absence of autocorrelation in error terms were checked in the data controls for multivariate analysis. Outlier distributions in the data were checked, and it was observed that all data forms were within the ± 2.5 limit, so data exclusion was not performed. Exploratory factor analysis (EFA) was applied to prepare the initial model for the scale, and confirmatory factor analysis (CFA) was applied to obtain the final model. After determining the scale model, reliability tests were performed using Cronbach’s α coefficient and test-retest analyses.

### Multivariate normal distribution

To verify multivariate normality, the Mahalanobis Distance criterion was applied to the data from the 106 participants, with all values falling within the ± 2.5 range [20]. The “Observations farthest from the centroid (Mahalanobis Distance)” feature in AMOS indicated an index value of 5.193, which is less than the threshold of 8, confirming multivariate normality [21]. With this assumption met, validity and reliability analyses were carried out.

## Results

### Demographic information of participants

The demographic information of the participants is presented in the table below (Table 2).

**Table 2:** Demographic Information of the Participants.

Variable	Groups		Frequency	Percent
**Gender**	**Female**		48	45.3
	**Male**		58	54.7
**Marital Status**	**Married**		67	63.2
	**Single**		39	36.8
**Title**	**Intern doctor**		51	48.1
	**Resident doctor**		1	0.9
	**General Practitioner**		16	15.1
	**Family Physician**		2	1.9
	**Medical Specialist**		17	16.0
	**Assistant Professor**		9	8.5
	**Associate Professor**		7	6.6
	**Professor**		3	2.8
**Specialty field**	**Intern doctor**		11	10.4
	**Internal Medicine**		74	69.8
	**Surgical Medicine**		18	17.0
	**Basic Medicine**		3	2.8
**Ethics Education Received**	**Yes**		65	61.3
	**No**		41	38.7
**Ethics Education**	**Undergraduate Education**		50	47.2
	**Course, Seminar, Symposium**		6	5.7
	**In-service training**		7	6.6
	**Doctorate Education**		2	1.9
	**No Education**		41	38.7
**Encountering Ethical Issues Status**	**Rarely**		21	19.8
	**Sometimes**		45	42.5
	**Very Often**		11	10.4
	**Quite Often**		26	24.5
	**Constantly**		3	2.8
**Receiving Ethical Consultancy Status**	**Yes**		9	8.5
	**No**		97	91.5
**Frequency of Moral Courage Encounters**	**Rarely**		20	18.9
	**Sometimes**		50	47.2
	**Very Often**		10	9.4
	**Quite Often**		25	23.6
	**Constantly**		1	0.9
**Total**			**106**	**100.0**
**Variable**		**Mean ± SD**		**Min – Max**
**Age**		37.39 ± 11.15		20-70
**Years of experience**		12.39 ± 11.08		1-46

SD; standard deviation.

### Construct validity

First, the Kaiser-Meyer-Olkin (KMO) test was conducted to assess the adequacy of the data for Exploratory Factor Analysis (EFA). T The KMO value indicates the suitability of the data for analysis, with a minimum value of 0.60 considered acceptable. Additionally, a high value for Bartlett’s Test of Sphericity supports the appropriateness of the data structure for the model [17]. The results of Bartlett’s Test of Sphericity calculated with the KMO test for the Moral Courage Scale are presented in the table below (Table 3).

**Table 3:** KMO and Bartlett’s test of sphericity.

Kaiser‒Meyer‒Olkin Measure of Sampling Adequacy.		0.900
**Bartlett’s Test of Sphericity**	**Approx. Chi-Square**	601.126
	**df**	36
	**p**	0.001*

df; degree of freedom, *p < 0,05; the test is statistically significant.

The KMO value was calculated as 0.900, and the Bartlett’s Test of Sphericity value was 601.126. A KMO test value of 0.90 indicates that the sample size is highly adequate for the scale. The sample size and structure were found to be suitable for the application of EFA [17].

The factor loadings, Corrected Item-total Correlations, and % Variance Explained obtained from the applied EFA are presented in the table below.

Factor loadings for the items in the scale were analyzed, considering a minimum threshold of 0.30. Upon examining the scale model, it was observed that there were no factor loadings below 0.30. The percentage of explained variance for the scale was calculated to be 73.6%, indicating that the explained variance ratio was at the desired level for the scale. The range of factor loadings varied between 0.453 and 0.850 (Table 4).

**Table 4:** Factor Loadings and Item–Total Correlations of the Moral Courage Scale.

Items	Factor Loadings
**Q1**	0.728
**Q2**	0.841
**Q3**	0.798
**Q4**	0.850
**Q5**	0.850
**Q6**	0.642
**Q7**	0.847
**Q8**	0.453
**Q9**	0.845
**% Variance Explained**	73.6

In the selection of the subdimensions, subdimensional ranges with eigenvalues above 1 and the varimax rotation method were used [22].

In EFA analyses, the number of factors is determined by different methods [23].

The first and most preferred criterion is the Kaisen criterion, and the roots (λ ≥ 1) greater than one in the covariance and correlation matrix are preferred.

The points where the slope starts to disappear in the number of factors determined by the Scree Plot Method are taken into consideration

Joliffe Criterion (taking as many factors as the number of eigenvalues greater than 0.7); as many factors as the number of eigenvalues 0.7 and greater (λ ≥ 7) are determined,

It is a practical method to determine as many factors as the number of eigenvalues greater than one,

Comprehensibility; selecting variables that can be explained by the structure of the variables,

The explained variance criterion; the number of factors is selected as the number of eigenvalues so that the cumulative variance explained by the eigenvalues is at least 67% (70%−95%), and it is necessary to determine the number of factors that will explain a very high variance.

There are different views on the explained variance criterion;

M to indicate the number of significant eigenvalues;


$$\sum {\mathrm{\backslash nolimits}}_{j=1}^{m}\frac{\lambda i}{p}\geq 2/3\;\;or\;\sum {\mathrm{\backslash nolimits}}_{j=1}^{m}\frac{\lambda i}{p}\geq 0.66$$

The smallest value of m satisfying the condition gives the number of significant principal components. On the other hand, while a cut-off point of 0.66 is considered appropriate by some authors, the cumulative variance ratio can be taken as 0.95 in science and natural sciences and 0.60 in social sciences where information is less precise [24].

In the selection of the subdimensions, subdimensional ranges with eigenvalues above 1 and the varimax rotation method were used [25].

Following the Exploratory Factor Analysis (EFA), the Confirmatory Factor Analysis (CFA) stage was conducted for the “Moral Courage Scale,” which consists of 9 items representing a single underlying dimension.

**Fig 1:**
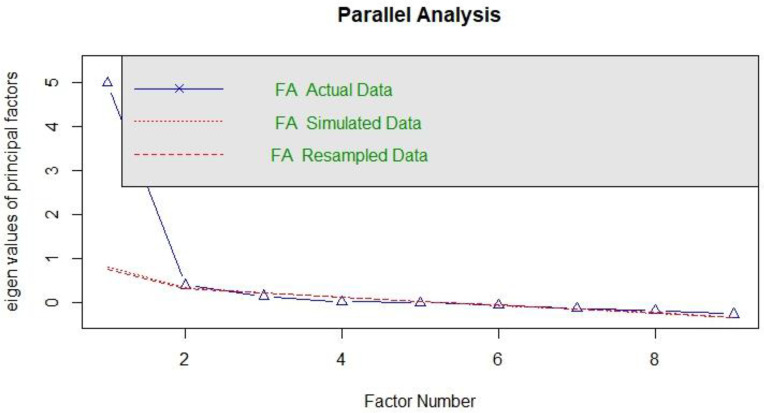
Factor structure obtained from parallel analysis.

The results of the parallel analysis confirming the unidimensional structure are presented in the following figure (Fig 1).

It was observed that, in the randomly generated data structures, the first factor exhibited an eigenvalue of sufficient magnitude [26].

### Confirmatory factor analysis

Confirmatory Factor Analysis (CFA) was conducted to test the accuracy of the scale model consisting of a single underlying dimension with 9 items obtained in the first stage of construct validity [27]. The initial structure of the scale was prepared and analysed. The goodness of fit indices obtained from the analysis were calculated as follows: χ2 (Chi-Square Goodness of Fit) = 76.730 and χ2/df = 2.842. The RMSEA (Root Mean Square Error of Approximation) value, used for sample adequacy, was found to be 0.132. While the NFI (Normed Fit Index) was 0.877, the CFI (Comparative Fit Index) and GFI (Goodness of Fit Index) were 0.915, and the IFI (Incremental Fit Index) was 0.917. It was observed that the NFI and RMSEA values did not fall within the desired range [28].

Due to the calculated values not meeting the desired level, modifications were required in the model, which may be due to relationships within the model, measurement errors, or existing relationships in the model that could not be analysed [29].

To apply modifications, covariances were added in pairs to the error terms of observable variables (scale items). Relationships between error terms within the same subscale were considered in covariance drawing. The main purpose of covariance drawing is to include abstract concepts explained simultaneously by two error terms without calculating them in the model [30]. The effects of external factors were included in the model through covariances, and modifications were made to the scale model. Items 5–7 and 8–9 contain similar expressions and are conceptually overlapping; this accounts for the correlation of measurement errors. The inclusion of error covariances in the model was employed to achieve a significant improvement in model fit. Relationships were established between the error terms with the highest modification indices [31].

**Fig 2:**
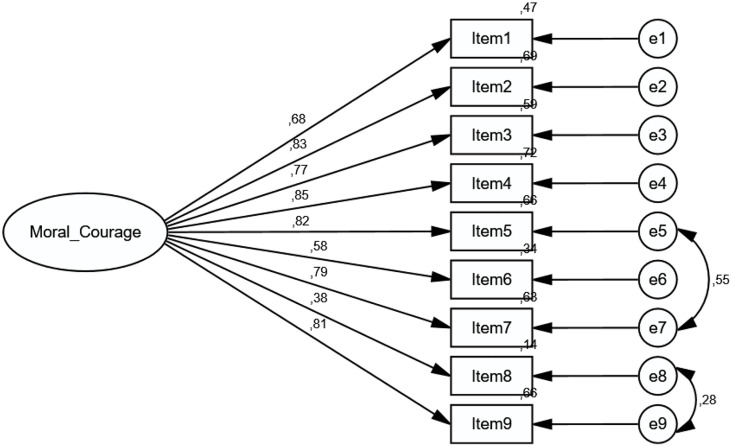
Diagram of the “Moral Courage Scale” in IBM SPSS AMOS 23 Program.

For the “Moral Courage Scale,” the highest residual terms, with modification index values, were e5-e7 and e8-e9, and covariances were drawn between these pairs of error terms in the model. The diagram of the final model of the scale is given below (Fig 2).

Confirmatory factor analysis (CFA) is one of the sub-analyses of structural equation modeling (SEM). In SEM analyses, multiple indices are provided and interpreted for model fit [32]. In the modified new model of the “Moral Courage Scale,” the χ2 value was found to be 39.904 and the χ2/df value was 1.596. The RMSEA value was calculated as 0.075, indicating that the sample size was sufficient for the scale model. It was also observed that the GFI value increased to 0.919, the CFI and IFI values increased to 0.975, and the NFI value increased to 0.936.

The χ2/df (χ2/sd ≤ 5) value decreased, and the RMSEA (RMSEA ≤0.08), GFI, CFI, NFI, and IFI values increased, indicating a good fit of the model. Due to the statistically significant and sufficient relationship established between the constructed SEM and the scales, the multiple group analysis stage was entered [31].

The single subscale 9-item scale model prepared with EFA for the “Moral Courage Scale” was confirmed by modifying the DFA model. Thus, the validity analysis of the “Moral Courage Scale” has been completed.

**Fig 3:**
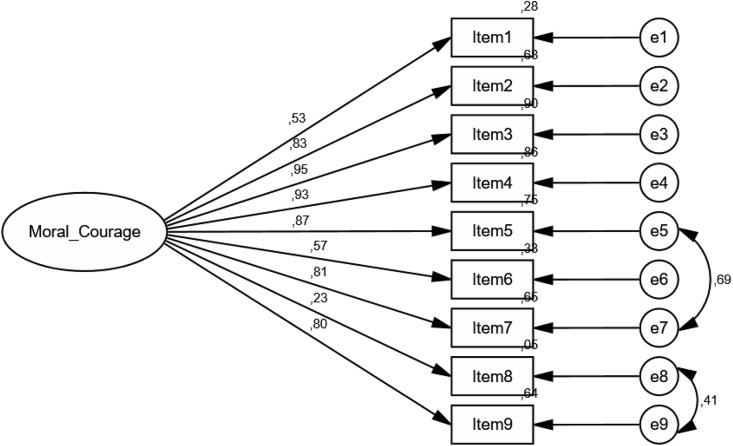
Diagram of the “Moral Courage Scale” for female.

**Fig 4:**
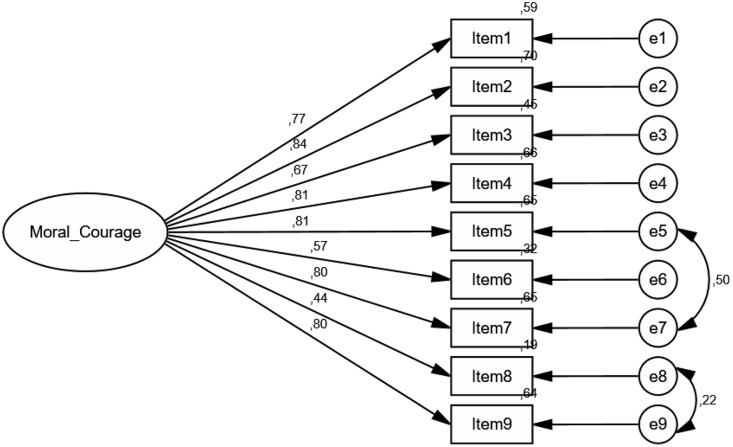
Diagram of the “Moral Courage Scale” for male.

Criterion-Related Validity, Group Comparisons, and Measurement Invariance The scale model was tested for measurement equivalence across female and male participants (Figs 3 and 4).

Upon examining the models, the χ²/df value was 1.611, indicating that the factor structure of the scale was equivalent for both female and male participants. Factor loadings were distributed according to the scale structure in both groups. The CFI value was 0.953, demonstrating structural equivalence. The RMSEA value of 0.077 indicates the adequacy of the sample size (Table 5).

**Table 5:** Evaluation results of structural equivalence and multi-group analysis models.

Model	χ2/df	CFI
**Unconstrained**	1.611	0.953
**Measurement weights**	1.585	0.948
**Measurement intercepts**	1.565	0.942
**Structural covariances**	1.552	0.943
**Measurement residuals**	2.180	0.900

The results of the structural, metric, scalar, and strict invariance comparisons are presented in Table 6.

**Table 6:** Results of structural, metric, and scalar invariance comparisons.

Model		CMIN	P
**Assuming model unconstrained to be correct**	**Measurement weights**	11.388	0.181
	**Measurement intercepts**	24.330	0.111
	**Structural covariances**	24.955	0.126
**Assuming model measuremet weights to be correct**	**Measurement intercepts**	12.943	0.165
	**Structural covariances**	13.567	0.194

The structural model was compared with the metric invariance model, and the difference was found to be statistically non-significant (p = 0.181). Accordingly, no differences were observed between the metric and structural models, indicating that the factor loadings of the scale items were equivalent across groups. Thus, the scale demonstrates metric invariance. Furthermore, when the metric model was compared with the scale model established for female participants, no statistically significant difference was found (p = 0.165). Similarly, comparison with the scale model established for male participants revealed no statistically significant difference (p = 0.194). Therefore, measurement invariance was confirmed for the Moral Courage Scale.

### Reliability

In order to calculate the reliability of the scale, Cronbach’s Alpha (α) coefficient of internal consistency and Split-Half method were used. The Cronbach’s α coefficient value ranges from 0 to 1, where values below 0.50 indicate that the scale is not reliable. As the coefficient value approaches 1, the reliability value also increases [33]. While increasing the number of items in the scale can increase the reliability value [27] in cases where the number of items is low, a Cronbach’s α coefficient value of 0.50 is considered acceptable [34]. Item-total correlation coefficients are also preferred for reliability. An item-total correlation coefficient value of at least 0.20 for items in a scale is considered sufficient for reliability [35].

The Cronbach’s α coefficient for the Moral Courage Scale was calculated as 0.91, and the range of total correlation coefficients for the items in the scale was found to be 0.387–0.797 (Table 7).

**Table 7:** Moral courage scale cronbach’s alpha (α) values.

Items	Corrected Item-Total Correlation	Cronbach’s Alpha if Item Deleted
**Q1**	0.660	0.878
**Q2**	0.770	0.872
**Q3**	0.705	0.874
**Q4**	0.773	0.870
**Q5**	0.749	0.872
**Q6**	0.553	0.887
**Q7**	0.756	0.871
**Q8**	0.387	0.916
**Q9**	0.797	0.869
**Cronbach’s α**	0.91	0.878

Item 8 had the lowest Corrected Item-Total Correlation, which was 0.387. This may be attributed to the 15-day interval between the test-retest analyses. The second stage of reliability analysis for the scale, which is the test-retest analysis, used a sample of 30 participants [36]. The results of the tests are presented in the table below (Table 8).

**Table 8:** Test-retest results.

Moral Courage Scale	Grup	Mean ± sd	Cronbach’s α	t	p^1^	r	p^2^
**Test**	56.81 ± 3.65	0.754	0.773	0.449	0.927	<0.001*
	**Retest**	56.55 ± 2.92	0.791			

sd; standard deviation, t; paired t test, r; Pearson correlation coefficient.

When the total score of the Moral Courage Scale was examined, a very high level of statistically significant positive relationship was found between the test and retest groups (p < 0.05).

The ICC values were calculated based on the test-retest analysis data. The results are presented in Table 9.

**Table 9:** Intraclass Correlation Coefficient (ICC).

Items	Intraclass Correlation	95% Confidence Interval	
		Lower Bound	Upper Bound
**Q1**	0.917	0.803	0.966
**Q2**	0.815	0.590	0.922
**Q3**	0.758	0.483	0.896
**Q4**	0.722	0.420	0.880
**Q5**	0.855	0.669	0.940
**Q6**	0.948	0.873	0.979
**Q7**	0.618	0.252	0.829
**Q8**	0.941	0.857	0.976
**Q9**	0.495	0.078	0.764

Examination of the items indicated that the ICC values for the test-retest results were at an adequate level [37].

### Scale score calculation

In the scale scoring, a 7-point Likert-type scoring was used for the responses. The maximum score that can be obtained from the scale is 63, while the minimum score is 7.

The values for the 106 participants in the study are given in the table below.

Of the 106 participants in the study, the lowest score obtained from the scale total was 11, and the highest score was 63, with a mean score of 54.43 ± 8.02 standard deviation (Table 10).

**Table 10:** Descriptive Statistics for the Moral Courage Scale Scores.

Moral Courage Scale	Mean ± sd	Min–Max Scores to Receive From the Scale
54.43 ± 8.02	11 - 63

sd; standard deviation.

## Discussion

Studies on moral courage directed towards physicians are quite limited. Understanding the level of moral courage of physicians from the pre-graduation period throughout their professional lives to prepare them for ethical problems they may encounter and to enable them to cope with these problems is of great importance. Although various views have been put forward regarding moral courage, there is no Turkish tool that measures these characteristics in physicians. Therefore, this study was conducted to evaluate the appropriateness, validity, and reliability of the Turkish version of the scale developed by Martinez (2016) to determine the level of moral courage of physicians. Conducting such a study for the first time in Türkiye is one of the strongest aspects of this research. The lack of research on the moral courage of physicians at the national and international levels limits the discussions on this subject. Physicians frequently encounter ethical challenges in their professional practice, which often leads to burnout [38]. This study makes an important contribution to understanding and improving physicians’ capacity to cope with ethical challenges.

A total of 106 participants were included in the study. The Kaiser-Meyer-Olkin (KMO) test value of the scale, which ensured multivariate normal distribution, was 0.90, indicating that the sample size was quite high in terms of evaluating the adequacy of the scale. The basic model of the scale, which formed the basis of the analyses, was prepared using exploratory factor analysis (EFA) and was confirmed by modifications made in the confirmatory factor analysis (CFA) model. Cronbach’s α coefficient was calculated to evaluate the internal consistency of the scale and found to be 0.91. Additionally, the range of total correlation coefficients for the items in the scale was calculated to be between 0.387 and 0.797. These results indicate that the scale was designed reliably and consistently and can be used.

In the analysis of the test-retest groups of the scale, statistically significant and highly positive relationships were found (p < 0.05). According to the results obtained with the 7-point Likert-type scoring used in the scale scoring, the maximum score that can be obtained from the scale was determined to be 63, while the lowest score was determined to be 7. The lowest score obtained from the scale total by the 106 participants included in the study was 11, the highest score was 63, and the mean score was found to be 54.43 ± 8.02 standard deviation. These results indicate that the scale is reliable and stable. It can be said that the participants’ levels of moral courage are generally high, and the scale successfully measures these differences.

In conclusion, this study includes the adaptation study of the Moral Courage Scale into Turkish, and the obtained data show that the scale can provide valid and reliable results. These results suggest that the scale can be an effective tool for evaluating the level of moral courage and can provide reliable results in studies. Additionally, the scale’s low number of items increases its usability and facilitates the answering of relevant items. Therefore, this scale will be able to determine the levels of moral courage of physicians, reveal cultural differences, and allow for the planning of necessary arrangements in both undergraduate and in-service trainings starting from the pre-graduation period, and the results obtained can offer solution proposals regarding the situation.

### Informed consent statement

Informed consent was obtained from all subjects involved in the study.

## Supporting information

S1 File Data collection form. (DOCX)
