# Supplementary material for: Cross-cultural adaptation, reliability, and validity of the Turkish version of moral courage scale for physicians
Source: PLoS One. 2025 Oct 30;20(10):e0333598. doi: 10.1371/journal.pone.0333598 (PMC12574860; doi:10.1371/journal.pone.0333598)
Supplement: S1 File — (DOCX) [file pone.0333598.s001.docx]

**Turkish Validity and Reliability of the Moral Courage Scale for Physicians Data Collection Form**

**Dear Participant,**

You are invited to participate in the research titled "Turkish Validity and Reliability of the Moral Courage Scale for Physicians." This study aims to examine the Turkish validity and reliability of the Moral Courage Scale for Physicians (MCSP). Participation in this research is entirely voluntary. You may choose not to participate or withdraw from the study at any time. The results of this research will be used for scientific purposes. If you withdraw from the study or are removed by the researcher, your data will not be used. However, once the data is anonymized, it will not be possible to withdraw from the study. All information collected from you will be kept confidential, and your identity will be protected when the research is published.

1. **Do you consent to participate in this study?**
   ( ) Yes
   ( ) No
2. **Your Age:**
3. **Your Gender:**
   Female ( ) Male ( )
4. **Your Marital Status:**
   Married ( ) Single ( ) Divorced ( )
5. **Your Title:**
   Intern Doctor ( )
   General Practitioner ( )
   Research Assistant ( )
   Specialist Doctor ( )
   Assistant Professor ( )
   Associate Professor ( )
   Professor ( )
6. **Please specify your field of expertise:**
7. **How many years have you been working?**
8. **Have you received any training in ethics?**
   ( ) Yes ( ) No
9. **If yes, please specify where you received the training:**
   ( ) Undergraduate Training
   ( ) In-Service Training
   ( ) Course/Seminar/Symposium, etc.
   ( ) Other:…………..
10. **Indicate how often you encounter ethical issues:**
    ( ) Never
    ( ) Rarely
    ( ) Sometimes
    ( ) Quite often
    ( ) Very often
    ( ) Always
11. **When faced with an ethical issue, did you seek ethical consultation (e.g., ethics committee)?**
    ( ) Yes ( ) No
12. **“Moral courage is defined as the ability to act according to one's moral and ethical values and beliefs, despite potential negative consequences for the individual, and the courage to adhere to one's ethical values and principles.”**

Indicate how often you encounter situations that require moral courage:
( ) Never
( ) Rarely
( ) Sometimes
( ) Quite often
( ) Very often
( ) Always

**Moral Courage Scale for Physicians (MCSP)**

|  | Strongly Disagree | Moderately  Disagree | Slightly Disagree | Neutral | Strongly Agree | Moderately  Agree | Slightly Agree |
| --- | --- | --- | --- | --- | --- | --- | --- |
| 1 I do what is right for my patients, even if I experience opposing social pressures (e.g., opposition from senior members of the healthcare team, medical guidelines, etc.). |  |  |  |  |  |  |  |
| 2 I use a guiding set of principles from my profession to help determine the right thing to do for my patients. |  |  |  |  |  |  |  |
| 3. My patients and colleagues can rely on me to exemplity moral behavior. |  |  |  |  |  |  |  |
| 4. I do what is right for my patients because it is the ethical thing to do. |  |  |  |  |  |  |  |
| 5. I go above and beyond what is required to do what is right for my patients. |  |  |  |  |  |  |  |
| 6 When faced with ethical dilemmas in patient care, I consider how both my professional values and my personal values apply to the situation before making decisions. |  |  |  |  |  |  |  |
| 7. When I do the right thing for my patients, my motives are pure. |  |  |  |  |  |  |  |
| 8. I do what is right for my patients, even if it puts me at risk( eg, legal risk, risk to reputation, etc.) |  |  |  |  |  |  |  |
| 9. I am determined to do the right thing for my patients. |  |  |  |  |  |  |  |
